# Editorial: Human machine interface-based neuromodulation solutions for neurorehabilitation

**DOI:** 10.3389/fnins.2022.987455

**Published:** 2022-08-25

**Authors:** Jing Wang, Jinhua Zhang, Haoyong Yu, Bin Shi

**Affiliations:** ^1^School of Mechanical Engineering, Xi'an Jiaotong University, Xi'an, China; ^2^Shaanxi Key Laboratory of Intelligent Robots, Xi'an Jiaotong University, Xi'an, China; ^3^Department of Biomedical Engineering, National University of Singapore, Singapore, Singapore

**Keywords:** transcranial magnetic stimulation (TMS), mechanism, neurorehabilitation, brain-computer interface (BCI), intermittent theta burst stimulation (iTBS)

Neurorehabilitation is a complex medical process which aims to aid recovery from a nervous system injury, and to minimize and/or compensate for any functional alterations resulting from nervous system injury disease. These diseases include stroke, spinal cord injury, cerebral palsy, Parkinson's disease, brain injury, and multiple sclerosis. Human-machine interface is a potential neuromodulation scheme for neurorehabilitation. To provide a platform for sharing the latest research findings in human machine interface-based neuromodulation for neurorehabilitation, we organized this Research Topic, in which 18 manuscripts have been accepted for publication, including 12 original research articles, four reviews, and two clinical trials. These articles stated that transcranial magnetic stimulation (TMS), brain–computer interface (BCI) and robots are the current advanced intervention techniques for the treatment of these diseases. In addition, the corresponding neural mechanisms were studied by healthy subjects using these intervention techniques. To a certain extent, these manuscripts have expanded the current understanding of diagnosis, treatment, and prognosis of such nervous system injury diseases.

According to different TMS stimulation pulses, TMS can be divided into three stimulation modes: single TMS (sTMS), double pulse TMS (pTMS), and repetitive TMS (rTMS). A study by Cai et al. explored that brain network activity modulates corticospinal excitability in 32 healthy individuals by recording electroencephalography (EEG) and single TMS measurements. The results suggested that corticospinal excitability can be modulated by the power spectrum of sensorimotor regions and the overall efficiency of functional networks. Thus, EEG network analysis can provide a useful complement to study the relationship between EEG oscillations and corticospinal excitability.

In addition to sTMS, rTMS technology is also widely used in the treatment of stroke, cerebral palsy, and multiple system atrophy (MSA). In studies focusing on recovery of dysphagia post-stroke, Yang, Cao, et al. and Xie Y.-l. et al. systemically evaluated the effect and safety of rTMS on recovery of dysphagia after stroke. They suggested that rTMS improved overall swallowing function and activity of daily living ability and reduced aspiration in post-stroke patients. Moreover, to explore the effect of 5 hz rTMS combined with wrist-ankle acupuncture on improving spasticity and motor function in children with spastic cerebral palsy by measuring electrophysiological parameters and behavior, 25 children with spastic cerebral palsy were enrolled in a single blind and randomized controlled trial. The authors found that wrist-ankle acupuncture combined with 5 Hz rTMS was the best for improving gross motor function and enhancing the conductivity of the corticospinal tract in children with cerebral palsy, but it could not highlight its clinical advantages in improving spasticity. Furthermore, executive dysfunction widely exists in a variety of neuropsychiatric diseases, and is closely related to the decline of daily living ability and function (Li J. et al.). Another study by Liu S. et al. analyzed the effect of low-frequency rTMS on executive function and its neural mechanism by using event-related potential (ERP). Thirty-one healthy subjects were randomly assigned to receive rTMS stimulations (1 Hz rTMS or sham rTMS) to the left dorsolateral prefrontal cortex (DLPFC) twice. They suggested that low-frequency rTMS of the left DLPFC can cause decline of cognitive flexibility in executive function, resulting in the change of N2 amplitude and the decrease of P3 and late positive component (LPC) components during task switching, which is of positive significance for the evaluation and treatment of executive function. In addition, MSA refers to a progressive neurodegenerative disease characterized by autonomic dysfunction, parkinsonism, cerebellar ataxia, as well as cognitive deficits. Zhang et al. systemically assessed the effects of Non-invasive brain stimulation (NIBS) on two subtypes of MSA: parkinsonian-type MSA (MSA-P) and cerebellar-type MSA (MSA-C). They found that NIBS can serve as a useful neurorehabilitation strategy to improve motor and cognitive function in MSA-P and MSA-C patients. However, they suggested that further high-quality articles are required to examine the underlying mechanisms and standardized protocol of rTMS as well as its long-term effect. Meanwhile, the effects of other NIBS subtypes on MSA still need further investigation.

Intermittent theta burst stimulation (iTBS) is a special form of repetitive transcranial magnetic stimulation (rTMS), which effectively increases cortical excitability and has been widely used as a neural modulation approach in stroke rehabilitation. A study by Ding et al. investigated the effects of iTBS on functional brain network through the resting-state EEG of stroke survivors. Thirty stroke survivors with upper limb motor dysfunction were studied. The authors provide evidence that iTBS modulates brain network functioning in stroke survivors. The acute increase in interhemispheric functional connectivity and overall efficiency after iTBS suggests that iTBS has the potential to normalize brain network function after stroke, which can be used for stroke rehabilitation. Furthermore, another study by Xie Y.-J. et al. explored the efficacy of cerebellar iTBS on the walking function of stroke patients. Thirty-six survivors with walking dysfunction who had suffered their first unilateral stroke were recruited. The authors found that applying iTBS over the contralesional cerebellum paired with physical therapy could improve walking performance in patients after stroke, suggesting that cerebellar iTBS intervention might be a non-invasive strategy to improve walking function for stroke survivors. Moreover, a study of Diao et al. investigated whether the individual level of GABA or NMDA receptor-mediated activity before stimulation is correlated with the after-effect in cortical excitability induced by iTBS. They found that that GABA_A_ receptor-mediated activity measured before stimulation is negatively correlated with the after-effect of cortical excitability induced by iTBS. The short-interval intracortical inhibitory (SICI) might be a good predictor of iTBS-induced LTP-like plasticity for a period lasting 15 min following stimulation.

A brain-computer interface (BCI) is a real-time communication system that connects the brain and external devices. The combination of BCI technology and hand rehabilitation robots is often used for motor function rehabilitation after stroke. In addition, some studies have explored cortical activation and neuroplasticity mechanisms in healthy subjects by BCI technology. Yang, Zhang, et al. provided medical evidence-based support for BCI in the treatment of upper limb dysfunction after stroke by conducting a meta-analysis of relevant clinical studies. A total of 13 randomized controlled trials involving 258 subjects were retrieved. The authors demonstrated that BCI training can effectively promote the recovery of upper limb motor function in stroke survivors, and the effect size was moderate. Moreover, Liu L. et al. evaluated the efficacy of BCI training in chronic stroke patients with moderate or severe paresis. Eighteen hospitalized chronic stroke patients with moderate or severe motor deficits participated. They demonstrated that BCI-based rehabilitation can effectively intervene in the motor performance of post-stroke patients with moderate or severe upper limb paralysis and is a potential strategy for stroke neurorehabilitation. The results shown that the functional connectivity between ipsilesional primary motor cortex (M_1_) and frontal cortex might be enhanced after BCI training. Furthermore, Li X. et al. studied the effectiveness of a post-stroke hand rehabilitation system, which is the sensorimotor rhythm (SMR)-based BCI with audio-cue, motor observation and multisensory feedback. Twenty-four stroke survivors with severe upper limb motor deficits were studied. The authors found that the hand rehabilitation system combined with conventional therapy may promote long-lasting upper limb motor improvement. In addition to BCI training for stroke subjects, Lin et al. analyzed effects of frequency of motor imagery (MI) BCI training on the central nervous system. Sixteen young healthy subjects were randomly assigned to a high frequency group with performed MI-BCI training once per day and low frequency group which performed once every other day. The results revealed that compared to the low frequency group, the high frequency group presents more cortical activation and better BCI performance. Meanwhile, the authors suggested that 30 min per day for five consecutive days may be the lowest effective dose of MI-BCI training to activate modulation of cortical activation in healthy subjects, which can be extrapolated in the future to stroke patients. Similarly, another study by Qiu et al. used synchronous functional near infrared spectroscopy (fNIRS) to analyze whether mirror visual feedback (MVF) and a soft robotic bilateral hand rehabilitation system have synergistic effects on cortical activation. Twenty healthy subjects were recruited to perform four different visual feedback tasks with simultaneous fNIRS monitoring. Four different visual feedback tasks include the real visual feedback (RVF) task, mirror visual feedback (MVF) task, bilateral robotic movement (BRM) task, and MVF + BRM task. The results found that the synergistic gain effect on cortical activation from mirror visual feedback combined with a soft robotic bilateral hand rehabilitation system for the first time, which could be utilized to guide the clinical application and the future studies. Furthermore, Liu Y. et al. conducted 4-week BCI-controlled supernumerary robotic finger (SRF) training in 10 right-handed subjects to study the neuroplasticity mechanisms. The results shown that cerebellar compensatory and inhibitory mechanisms exist during BCI-controlled SRF training, and this result provides evidence for the neuroplasticity mechanism brought about by BCI-controlled motor-augmentation devices.

Muscle synergies have been largely used in many application fields, including motor control studies, prosthesis control, movement classification, rehabilitation, and clinical studies. Zhao et al. analyzed the performance of five methods for the extraction of spatial muscle synergy, namely, principal component analysis (PCA), independent component analysis (ICA), factor analysis (FA), non-negative matrix factorization (NMF), and AEs using simulated data and a publicly available database. The results showed that the performance of synergy extraction methods was affected by the noise and the number of channels, and classification algorithms were sensitive to the extraction methods. Moreover, the effect and mechanism of underlying enriched rehabilitation as a potentially effective strategy to improve gait and cognitive performance in patients with early Parkinson's disease (PD) were explored (Wang et al.). The enriched rehabilitation represents that the enriched sensorimotor environmental stimulation paired with different types of sensory and motor exercises was applied to improve gait disorder and cognitive function in PD. The authors found that enriched rehabilitation could serve as a potentially effective therapy for early-stage PD for improving gait performance and cognitive function. The underlying mechanism based on functional magnetic resonance imaging (fMRI) involved strengthened resting-state functional connectivity (RSFC) between the left dorsolateral prefrontal cortex (DLPFC) and other brain regions.

After the analysis of 18 manuscripts in this Research Topic, we can draw the following conclusions. The rTMS could improve overall swallowing function and activity of daily living ability in post-stroke patients. Furthermore, rTMS can serve as a useful neurorehabilitation strategy to improve motor and cognitive function in MSA-P and MSA-C patients. However, further studies are performed to examine the underlying mechanisms and standardized protocol of rTMS as well as its long-term effect. The iTBS has the potential to normalize brain network function after stroke and also might be a noninvasive strategy to improve walking function for stroke survivors. The BCI-based rehabilitation can effectively intervene in the motor performance of post-stroke patients with moderate or severe upper limb paralysis and is a potential strategy for stroke neurorehabilitation.

## Author contributions

BS was responsible for drafting the manuscript. JW revised the manuscript. JZ and HY analyzed and discussed the results. All authors read and approved the final manuscript.

## Conflict of interest

The authors declare that the research was conducted in the absence of any commercial or financial relationships that could be construed as a potential conflict of interest.

## Publisher's note

All claims expressed in this article are solely those of the authors and do not necessarily represent those of their affiliated organizations, or those of the publisher, the editors and the reviewers. Any product that may be evaluated in this article, or claim that may be made by its manufacturer, is not guaranteed or endorsed by the publisher.

